# Effects of Long-Term Paired Associative Stimulation on Strength of Leg Muscles and Walking in Chronic Tetraplegia: A Proof-of-Concept Pilot Study

**DOI:** 10.3389/fneur.2020.00397

**Published:** 2020-05-20

**Authors:** Andrei Rodionov, Sarianna Savolainen, Erika Kirveskari, Jyrki P. Mäkelä, Anastasia Shulga

**Affiliations:** ^1^BioMag Laboratory, HUS Medical Imaging Center, University of Helsinki and Helsinki University Hospital, Helsinki, Finland; ^2^Clinical Neurosciences, Clinical Neurophysiology, HUS Medical Imaging Center, University of Helsinki and Helsinki University Hospital, Helsinki, Finland; ^3^Clinical Neurosciences, Neurology, Helsinki University Hospital, Helsinki, Finland

**Keywords:** paired associative stimulation, walking, TMS, spinal cord injury, neuroplasticity

## Abstract

Recovery of lower-limb function after spinal cord injury (SCI) is dependent on the extent of remaining neural transmission in the corticospinal pathway. The aim of this proof-of-concept pilot study was to explore the effects of long-term paired associative stimulation (PAS) on leg muscle strength and walking in people with SCI. Five individuals with traumatic incomplete chronic tetraplegia (>34 months post-injury, motor incomplete, 3 females, mean age 60 years) with no contraindications to transcranial magnetic stimulation (TMS) received PAS to one or both legs for 2 months (28 sessions in total, 5 times a week for the first 2 weeks and 3 times a week thereafter). The participants were evaluated with the Manual Muscle Test (MMT), AIS motor and sensory examination, Modified Asworth Scale (MAS), and the Spinal Cord Independence Measure (SCIM) prior to the intervention, after 1 and 2 months of PAS, and after a 1-month follow-up. The study was registered at clinicaltrials.gov (NCT03459885). During the intervention, MMT scores and AIS motor scores increased significantly (*p* = 0.014 and *p* = 0.033, respectively). Improvements were stable in follow-up. AIS sensory scores, MAS, and SCIM were not modified significantly. MMT score prior to intervention was a good predictor of changes in walking speed (${\text{R}}_{\text{adj}}^{2}$ = 0.962). The results of this proof-of-concept pilot study justify a larger trial on the effect of long-term PAS on leg muscle strength and walking in people with chronic incomplete SCI.

## Introduction

Improving mobility in individuals with chronic spinal cord injury (SCI) remains a major clinical challenge (1, 2). Recovery of walking after SCI varies considerably and depends on e.g., age and severity of injury (3). People with incomplete lesions have the greatest probability to regain movement in the lower limbs as they have residual synaptic connectivity in the corticospinal tracts. It is known that spontaneous recovery is limited in all SCI subpopulations and usually occurs during the first 3 months and reaches a plateau by 9 months post-injury (4).

It is important to develop non-invasive neuromodulation for restoration of locomotor function (1, 3, 5, 6). Paired associative stimulation (PAS) specifically targets residual synaptic connections and may be effective in enhancing motor control over selected weak muscles. PAS combines transcranial magnetic stimulation (TMS) of the motor cortex and peripheral nerve stimulation (PNS) (7, 8). One PAS consists of a TMS pulse delivered to the cortical target at a predefined timing interval with the PNS given to a corresponding contralateral nerve. Repeated TMS-PNS pairing can induce durable changes in the cortico-cortical (7) and the corticospinal-motoneuronal synapses, (9) referred to as long-term potentiation (LTP)-like plasticity (10, 11). The potential of PAS to increase synaptic strength at the spinal level has a clear theoretical therapeutic significance for SCI and provides a possibility for returning motor control over paralyzed muscles.

Long-term application of PAS with the development of novel stimulation protocols has shown promising results in rehabilitation after SCI (12–15). This protocol reinforces corticospinal transmission at a wide range of interstimulus intervals (ISIs) between TMS and PNS, plausibly due to an increased number of interactions between pre- and post-synaptic volleys (12). High-intensity TMS pulses (100% of maximum stimulator output [MSO]) employed in the protocol generate multiple descending volleys, and high-frequency trains of PNS (50–100 Hz) increase the number of antidromic volleys. The method is superior to PNS in its ability to improve hand function in tetraplegic individuals (16). Long-term PAS is suggested to induce long-term potentiation (7) and to possibly contribute to functional reorganization in the corticospinal tracts (9), which augments motor input to the paralyzed muscles and leads to increased muscle strength and corresponding limb function (12, 14, 17).

Several studies reported changes in the excitability of corticospinal projections to the lower limb muscles by means of PAS (18–21). Reports on long-standing functional recovery by means of long-term PAS are sparse. Only one case study reported regained plantarflexion and dorsiflexion of the ankles of both legs in an individual with paraplegia after long-term PAS (14). Thus, long-term PAS-induced functional outcomes essential for walking improvement remain generally unexplored.

Muscle strength is strongly correlated to functional gait outcomes (22, 23). Weakness in individual muscles modifies walking and induces different variations of pathological gait patterns (24). Walking alteration after SCI depends on weakness in key muscles important for gait, their residual strength, and the availability of muscles to compensate for this weakness. Thus, long-term PAS may be an important therapeutic option for specific targeting of the weakest muscles and for enhancing their strength, thereby improving walking in a heterogenic SCI population. Here, we investigated the effects of a long-term PAS intervention of 8 weeks on leg muscle strength and walking ability. Our primary goal was to assess whether long-term PAS delivered 3–5 times per week to the nerves supplying the weakest muscles for 20 min per nerve can improve motor function as measured by the Manual Motor Test.

## Materials and Methods

### Participants

Five individuals (3 females, age range 48–70 years, mean age 60) with chronic incomplete tetraplegia of traumatic origin (Table 1) were enrolled in the study. The study was registered at clinicaltrials.gov (NCT03459885). Inclusion criteria were cervical incomplete SCI of traumatic origin and age 18–70 years. Exclusion criteria were contraindications for TMS or Magnetic Resonance Imaging (MRI), including the presence of intracranial metal objects, implanted electronics, epilepsy, pregnancy, high intracranial pressure, and any brain pathology visible in computer tomography or MRI (25). Initial leg muscle strength and walking ability varied between participants (Table 1). Conventional rehabilitation and medication of participants prior to the study were not modified during the intervention or the follow-up (Table 1). To ensure that any lack of vitamins or minerals would not hinder the therapeutic effect, all participants were asked to take a standard multivitamin dose during intervention and follow-up. Participants provided written informed consent prior to the study. The study was approved by the Ethics Committee of the Hospital District of Helsinki and Uusimaa.

**Table 1:** Patient characteristics.

**Patient**	**Age**	**Neurological level**	**AIS**	**Time since injury (years, months)**	**Conventional rehabilitation (min x times per week)**	**Medication affecting CNS (mg/day)**	**MMT score L/R and walking before the study**
1	60–65	C1	D	3 y, 2 m	None	tizanidine (4–10)	30/38—ambulatory
2	55–60	C5	D	3 y, 0 m	physiotherapy (60 × 1), gym (60 × 2), swimming pool (60 × 1), walking training (60 × 1)	pregabalin (150), tizanidine (12), baclofen (50)	35/26—ambulatory
3	70–75	C1	D	2 y, 10 m	physiotherapy (60 × 2), gym (90 × 3), swimming pool (60 × 2), standing with weight support (60 × 7), assisted cycling (60 × 7)	amitriptylline (25), gabapentin (1500), clonazepam (1), baclofen (60)	18/14—non-ambulatory
4	45–50	C5	D	12 y, 2 m	physiotherapy (60 × 2), gym (60 × up to 6), swimming pool (45 × 1), cycling on adapted bicycle (120–180 × 2–3)	zopiclone (7.5), baclofen (20)	26/8—ambulatory
5	60–65	C5	D	8 y, 8 m	physiotherapy (60 × 1), swimming pool (45 × 1)	none	43/32—ambulatory

*AIS, American Spinal Injury Association Impairment Scale; D, motor incomplete SCI with half or more of key muscle functions below the neurological level having a muscle grade ≥ 3; CNS, central nervous system*.

### Study Design

**Figure 1:**
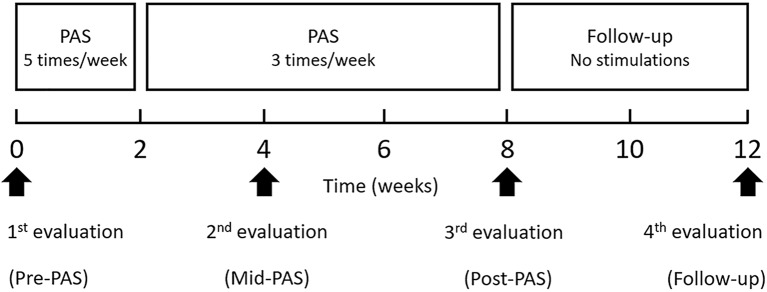
Time course of intervention.

PAS was given for 2 months, 5 times per week during first 2 weeks (10 sessions) and 3 times per week during the subsequent interventions (18 sessions, Figure 1). The duration of one PAS session depended on the number of stimulated nerve-hotspot pairs and lasted 80–120 min (Table 2). Nerves supplying the weak muscles with MMT scores of 0 to 3 (pre-PAS) were selected for stimulation in each patient. This focused the treatment on the weakest muscles and optimized duration of a single PAS session. Thus, in participant 1, only the nerves of the left leg were stimulated, in participants 2 to 4 both legs were stimulated, and in participant 5 only the right leg was stimulated. All stimulated nerves are shown in Table 2. The patients were evaluated clinically immediately prior to the study (pre-PAS), after 1 month of stimulation (mid-PAS), after 2 months of stimulation (post-PAS), and after the 1-month follow-up. All enrolled patients completed the study. No patients were lost to follow-up and the data of all patients were analyzed.

**Table 2:** Stimulation parameters.

		**Tibial nerve**		**Peroneal nerve**		**Femoral nerve**		**Gluteal nerve**		
**Participant**	**Stimulated legs (number of nerves/Time of stimulation per session/per participant)**	**ISI left/right**	**PNS intensity left/right**	**ISI left/right**	**PNS intensity left/right**	**ISI left/right**	**PNS intensity left/right**	**ISI left/right**	**PNS intensity left/right**	**Local anesthetic**
	**Min**	**ms**	**mA**	**ms**	**mA**	**ms**	**mA**	**ms**	**mA**	
1	Left (4/80/2240)	0/–	12/–	1/–	20/–	−15/–	35/–	0/–	45/–	No
2	Both (6/120/3360)	–/1	–/30*	–/1	–/46*	−6/-6	33/33*	−13/-16	20/20*	Gluteal
3	Both (6/120/3360)	3/4	21/22	–/–	–/–	19/22	9/21	14/13	50/80*	All
4	Both (6/120/3360)	–/16	–/10	–/8	–/20	−17/-15	25/10	−4/4	80/80*	Gluteal
5	Right (4/80/2240)	–/13	–/35*	–/13	–/39*	–/0	–/35*	–/0	–/40*	No

ISI, interstimulus interval; PNS, peripheral nerve stimulation; *, intensity was gradually increased during a stimulation session or from session to session; –, not stimulated.

### Navigated Transcranial Magnetic Stimulation (nTMS)

Biphasic TMS pulses were delivered to the motor cortex by an Eximia magnetic stimulator (Nexstim Ltd., Finland) with a figure-of-eight coil (outer diameter 70 mm). The patient's structural T1-weighted MRI (obtained with a 3T Siemens Verio scanner, Siemens Healthcare, Germany) was employed in NBS System 4.3 to build an individual 3D head model. Gyral anatomy was utilized to identify a TMS hotspot, a site within the contralateral motor cortex where stimulation most readily elicited MEPs from the muscle of interest. The hotspots were defined for the (first and second choice, respectively) abductor hallucis or soleus muscles (innervated by the tibial nerve), tibialis anterior or extensor digitorum brevis muscles (peroneal nerve), vastus medialis or lateralis muscles (femoral nerve), and gluteus maximus (gluteal nerve) using a built-in Eximia EMG device and surface electrodes (Neuroline 720; Ambu A/S, Baller- up, Denmark). If no MEPs were obtained from the first-choice muscle, the second-choice muscle was used. The stimulated hotspots in each participant corresponded to stimulated nerves (Table 2); e.g., in participant 1, four hotspots in the left hemisphere were identified corresponding to muscles innervated by right tibial, peroneal, femoral, and gluteal nerves. Resting motor thresholds (RMTs) were higher than 100% of MSO; consequently, a weak voluntary muscle preactivation was used to obtain MEPs. TMS with an intensity of 100% of MSO was used to elicit reliable MEPs during motor mapping, and the same TMS intensity was thereafter used for all PAS sessions.

### Peripheral Nerve Stimulation (PNS)

The Dantec Keypoint electroneuromyography device (Natus Medical Inc., USA) and surface electrodes utilized for MEP recordings were used to determine mean latency, amplitude, and persistence of F-waves. For this purpose, 10 responses to 0.2-ms pulses at supramaximal intensity were recorded from the same muscles as in MEP measurements in each subject. PNS intensity used for PAS was defined for each participant individually as the minimum intensity inducing F-responses to single 1-ms pulses (14, 16).

During PAS, 100-Hz PNS trains (1-ms square pulses, 6 pulses per train, train duration 100 ms) (15) were delivered using the Keypoint device and surface electrodes. For the gluteal nerve stimulation, the electrode placement was determined by an anatomical landmark centered at the ischial tuberosity (26). To ensure appropriate gluteal nerve stimulation, a tape roll (45 × 25 mm) was attached on top of the electrodes and the patient sat on it pressing the electrodes toward the nerve. For the femoral nerve stimulation, crossing of the inguinal crease and femoral artery, located along the course of the femoral nerve, was selected for electrode placement (27). Constant pressure was manually applied to the electrodes to ensure femoral nerve activation. The tibial nerve was stimulated behind the medial malleolus. The peroneal nerve was stimulated at the ankle.

### Paired Associative Stimulation (PAS)

In all PAS sessions, TMS intensity was 100% of stimulator output (SO) and PNS intensity was defined individually for each peripheral nerve. The conduction times for lower motor neurons and upper motor neurons were estimated individually for each hotspot-nerve pair by measuring the minimum F latency and average MEP latency. ISI between TMS and PNS were calculated using the formula F-latency minus MEP latency (13) to coincide with the induced neuronal volleys at the level of the lumbar spinal cord. Thus, ISI was individually adjusted and varied between participants, depending on their physiological measures and the extent and location of the injury. A positive ISI indicates that PNS precedes TMS; a negative ISI indicates that TMS precedes PNS. For PAS, the participants were seated in a comfortable chair (14, 16). One PAS session consisted of stimulations of 4 to 6 hotspot-nerve pairs delivered in a pseudo-random order (20 min or 240 TMS-PNS pairs, see Table 2 for stimulated nerves and stimulation time per patient). The participants were instructed to plantarflex the ankle and slightly flex the knee during tibial nerve stimulation (10 min per movement), dorsiflex the ankle during peroneal nerve stimulation, slightly flex the hip and extend the knee (10 min per movement) during femoral nerve stimulation, and slightly extend the hip (by pressing the whole leg down in sitting position) and abduct the hip during gluteal nerve stimulation (10 min per movement).

All patients tolerated TMS well. All patients were offered local skin anesthesia by EMLA ointment to reduce possible uncomfortable sensations caused by PNS (12, 28). Three patients chose to use this ointment (Table 2).

### Clinical Evaluations

An experienced physiotherapist specialized in SCI assessed the strength of gluteus maximus and medius, iliopsoas, quadriceps femoris, tibialis anterior, tibialis posterior, peroneus, long toe extensors, hamstrings, gastrocnemius, and hip adductors with the Manual Muscle Test (MMT). Spasticity was assessed with the Modified Ashworth Scale (MAS) from the hip (flexors, extensors, adductors), knee (flexors, extensors), and ankle (plantar flexors) muscles. Motor function was also evaluated with the standard American Spinal Injuries Association Impairment Scale (AIS). The physiotherapist did not participate in PAS and was blinded to the rule of nerve selection. The physiotherapist also measured gait function. For this purpose, the maximum distance that patients were able to walk without a break and without assistance of another person and the corresponding total time were measured and used to calculate mean walking speed. This test was selected to evaluate the functional outcome of PAS. MMT, AIS examination, and walking test were performed at each evaluation. A physician examined the patients' sensory function with AIS. The MAS, AIS examination of sensory function, and Spinal Cord Independence Measure (SCIM) were performed at the beginning of the intervention and at follow-up.

### Statistical Analysis

The mean MMT score of all evaluated muscles in both legs and the mean MMT scores of the muscles innervated by each of the stimulated nerves (partial MMT) in both legs were calculated in each participant for each evaluation separately. The muscles with a value of 5 before the intervention (corresponding to full muscle strength) were excluded from the analysis as no further improvement could be detected by MMT. The differences between MMT scores obtained in Mid- and Pre-PAS (change during the first month of intervention), Post- and Pre-PAS (change during the entire intervention), and Follow-up and Pre-PAS (change during the intervention and the follow-up) were also calculated. The mean AIS motor scores in both legs were computed in each participant for each evaluation separately, excluding the muscles with a value of 5 before the intervention. Differences between mean AIS scores were computed as for calculations for MMT scores. Differences in walking speed in pre-PAS and post-PAS and in pre-PAS and follow-up were computed. Due to technical failure, the walking test was not performed for patient 1 in mid-PAS and the results of the second walking evaluation were not included in the group analysis. The sum of points obtained with the MAS was calculated for each of the four evaluations separately. The sum of AIS sensory scores and SCIM was computed for the pre-PAS and the follow-up.

Statistical comparisons were performed in IBM SPSS Statistics (IBM Corp, Armonk, NY, USA, Version 24.0.) using the Friedman test and Wilcoxon signed-rank test where appropriate. Results are reported as medians. In addition, mean and standard error of the mean are shown in the tables and quartiles in figures. Regression analysis was used to assess the extent to which the total variation in the changes of walking speed (dependent variable) in the follow-up can be explained by the MMT score in the pre-PAS (independent variable). Three regressions were computed separately for all MMT scores, MMT scores of key muscles, and MMT scores of remaining muscles. Key muscles included gluteus maximus and gluteus medius, knee flexors, knee extensors, and ankle plantar flexors. This selection was based on previous reports on their significance particularly for walking speed improvement (22) and neuromotor strategies used in human locomotion (23). The remaining muscles included hip flexors, ankle dorsiflexors, long toe extensors, and adductors. The relationship between MMT scores and changes in walking speed were assessed with Spearman's rank correlation coefficients.

## Results

**Figure 2:**
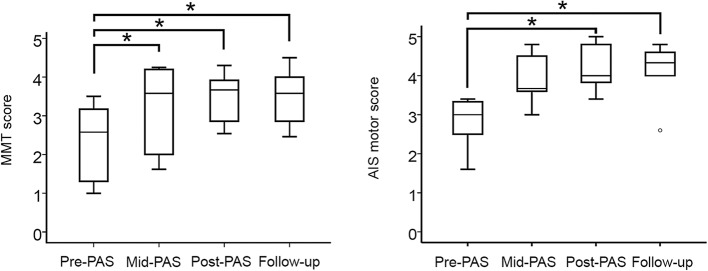
Medians of MMT scores **(left)** and AIS motor scores **(right)** before the intervention (Pre-PAS), after 1 month (Mid-PAS), and after 2 months (Post-PAS) of stimulations and in the 1-month follow-up. Asterisks show significant differences (*n* = 5, *p* < 0.05).

Total MMT scores were increased by the PAS from Md = 2.58–3.58 [*n* = 5, χ^2^(3) = 10.563; *p* = 0.014, Friedman's test]. *Post-hoc* analysis with Wilcoxon signed-rank test revealed an increase of total MMT scores in Mid-PAS (see Figure 2 and Table 3, *n* = 5, Md = 0.69, *z* = −2.023; *p* = 0.043), Post-PAS (*n* = 5, Md = 1.23, *z* = −2.023; *p* = 0.043), and follow-up (*n* = 5, Md = 1.15, *z* = −2.023; *p* = 0.043) when compared with Pre-PAS. Partial MMT scores were increased by the PAS from Md = 2.40–3.67 [*n* = 5, χ^2^(3) = 11.298, *p* = 0.01, Friedman's test]. *Post-hoc* analysis with Wilcoxon signed-rank test revealed an increase of partial MMT scores in Mid-PAS (Table 3, *n* = 5, Md = 0.60, *z* = −2.023, *p* = 0.043), Post-PAS (*n* = 5, Md = 1.10, *z* = −2.023, *p* = 0.043) and follow-up (*n* = 5, Md = 1.10, *z* = −2.023, *p* = 0.043) when compared with Pre-PAS. Raw MMT scores are presented in Supplementary Table 1.

**Table 3:** Manual muscle test (MMT) scores.

**Patient**	**MMT average score (all muscles)**				**MMT difference (all muscles)**			**MMT average score (stim)**				**MMT difference (stim)**		
	**Pre-PAS**	**Mid-PAS**	**Post-PAS**	**Follow-up**	**Mid-PAS—**	**Post-PAS—**	**Follow-up—**	**Pre-PAS**	**Mid-PAS**	**Post-PAS**	**Follow-up**	**Mid-PAS—**	**Post-PAS—**	**Follow-up—**
					**Pre-PAS**	**Pre-PAS**	**Pre-PAS**					**Pre-PAS**	**Pre-PAS**	**Pre-PAS**
1	3.17	3.58	3.67	3.58	0.42	0.50	0.42	3.00	3.50	3.67	3.67	0.50	0.67	0.67
2	2.58	4.25	3.92	4.00	1.67	1.33	1.42	2.40	4.20	3.90	3.90	1.80	1.50	1.50
3	1.31	2.00	2.54	2.46	0.69	1.23	1.15	1.30	1.90	2.40	2.40	0.60	1.10	1.10
4	1.00	1.62	2.86	2.86	0.62	1.86	1.86	0.91	1.50	2.82	2.64	0.60	1.91	1.73
5	3.50	4.20	4.30	4.50	0.70	0.80	1.00	3.38	4.00	4.13	4.38	0.63	0.75	1.00
Median	2.58	3.58	3.67	3.58	0.69	1.23	1.15	2.40	3.50	3.67	3.67	0.60	1.10	1.10
Mean	2.31	3.13	3.46	3.48	0.82	1.14	1.17	2.20	3.02	3.38	3.40	0.83	1.19	1.20
SE	0.50	0.56	0.33	0.37	0.22	0.23	0.24	0.48	0.55	0.33	0.38	0.24	0.23	0.19

*MMT average score (all muscles) and MMT average score (stim)—means of MMT scores calculated for all evaluated muscles in each participant and for the muscles innervated by the stimulated nerves in each participant, respectively*.

The median AIS motor scores increased from 3.00 (pre-PAS) to 4.33 (Follow-up) [*n* = 5, χ^2^(3) = 8,733; *p* = 0.033; Friedman's test]. *Post-hoc* analysis with Wilcoxon signed-rank test revealed an increase of AIS motor scores in Post-PAS (see Figure 2 and Table 4, *n* = 5, Md = 1.40, z = −2.023; *p* = 0.043) and in follow-up (*n* = 5, Md = 1.40, *z* = −2.032; *p* = 0.042) when compared with Pre-PAS. The differences between Mid-PAS vs. Pre-PAS (*n* = 5, Md = 1.17, *z* = −1.841; *p* = 0.066) were not statistically significant. Raw AIS motor scores are presented in Supplementary Table 2.

**Table 4:** AIS motor scores.

**Patient**	**Average motor score**				**Difference**		
	**Pre-PAS**	**Mid-PAS**	**Post-PAS**	**Follow-up**	**Mid-PAS—Pre-PAS**	**Post-PAS—Pre-PAS**	**Follow-up— Pre-PAS**
1	3.40	4.80	4.80	4.80	1.40	1.40	1.40
2	3.33	4.50	4.00	4.33	1.17	0.67	1.00
3	2.50	3.67	3.83	4.00	1.17	1.33	1.50
4	1.60	3.60	3.40	2.60	2.00	1.80	1.00
5	3.00	3.00	5.00	4.60	0.00	2.00	1.60
Mean	2.77	3.91	4.21	4.07	1.15	1.44	1.30
SE	0.33	0.33	0.30	0.39	0.32	0.23	0.13

*AIS, American Spinal Injury Association Impairment Scale*.

Walking speed did not change at the group level across all evaluations [Md = 0.31 m/s [Pre-PAS] and 0.35 m/s [Follow-up], *n* = 4, χ^2^(2) = 3,500, *p* = 0.174; Friedman's test]. Nevertheless, walking speed increased in the post-PAS in all ambulatory participants (*n* = 5, *p* = 0.031, binomial test). Walking speed increased post-PAS in participants 1, 2, 4, and 5 (Table 5, *n* = 4, Md = 0.10 m/s). Participant 3, who was non-ambulatory before the intervention, could take several steps with an Eva support walker but this result was not quantifiable. Walking distance increased in 3 out of 4 ambulatory participants (Table 5). Changes in walking speed were heterogenic in follow-up. Participants 2 and 5 further improved their walking speed, whereas participant 3 remained stable. Although the walking speed of participant 1 decreased slightly in the follow-up when compared with post-PAS speed, the speed was still higher than in the pre-PAS test (Table 5). Participant 4 (with the longest post-trauma time) demonstrated a more pronounced decrease of walking speed in the follow-up.

**Table 5:** Walking speed.

	**Distance (m)/Time (s)**			**Speed (m/s)**			**Difference**	
**Patient**	**Pre-PAS**	**Post-PAS**	**Follow-up**	**Pre-PAS**	**Post-PAS**	**Follow-up**	**Post-PAS—Pre-PAS**	**Follow-up— Pre-PAS**
1	80/266	86/197	172/435	0.30	0.44	0.40	0.14	0.09
2	43/205	430/1560	125/420	0.21	0.28	0.30	0.07	0.09
3	n/a	n/a	n/a	n/a	n/a	n/a	n/a	n/a
4	38/117	27/60	38/144	0.32	0.45	0.26	0.13	−0.06
5	86/107	420/494	688/689	0.80	0.85	1.00	0.05	0.20
Median	61.50/161.00	253.00/345.50	148.50/427.50	0.31	0.45	0.35	0.10	0.09
Mean	61.75/173.75	240.75/577.75	255.75/422.00	0.41	0.50	0.49	0.09	0.08
SE	11.70/33.83	95.77/303.85	131.24/99.58	0.12	0.11	0.15	0.02	0.05

*n/a, not available*.

**Figure 3:**
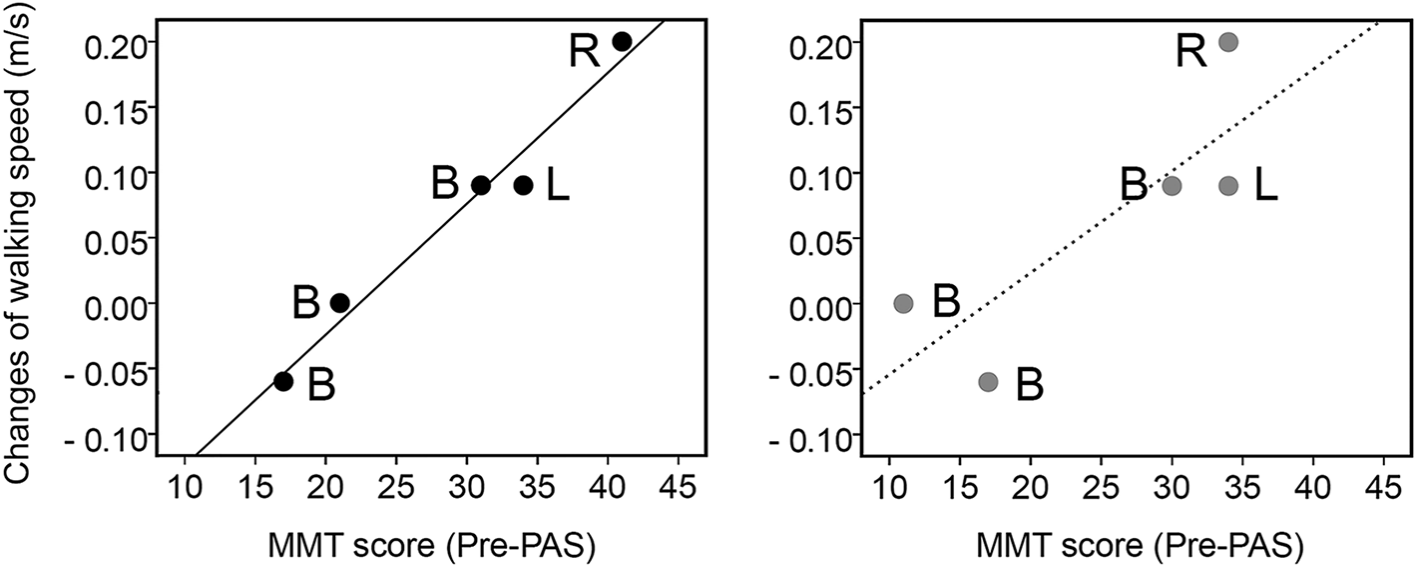
Relationship between the sum of the MMT scores collected in stimulated lower limbs prior the intervention (Pre-PAS) from the key muscles **(left)** and remaining muscles **(right)** and changes in walking speed obtained during follow-up. Linear regression—solid and dashed lines. R—only right leg was stimulated, L—only left leg was stimulated, B—both legs were stimulated.

A significant regression equation was found for all MMT scores [*n* = 5, *F*_(1, 3)_ = 18.564, *p* = 0.024, *r* = 0,872, *p* = 0.054], with an *R*^2^ of 0.861. The patients' predicted walking speed was equal to −0.187 + 0.005x (total MMT score) m/s. The adjusted $R_{\text{adj}}^{2}$ = 0.815 indicates that ~81.5% of variance in changes of walking speed can be explained by the initial total MMT score. When MMT scores of key muscles were used for prediction of changes in walking speed, a significant regression equation *y* = −0.224 + 0.010x (MMT score of key muscles) was observed for the key muscle MMT score [*n* = 5, *F*_(1, 3)_ = 102,330, *p* = 0.002] with an *R*^2^ of 0.972 (adjusted $R_{\text{adj}}^{2}$ = 0.962). The MMT score of key muscles in pre-PAS and changes of walking speed in the follow-up were linearly related (Figure 3) and highly correlated (*r* = 0.975, *p* = 0.005). Results of regression analysis with MMT scores of the remaining muscles were not significant [Figure 3B, *n* = 5, *F*_(1, 3)_ = 6.608, *p* = 0.082, adjusted $R_{\text{adj}}^{2}$ = 0.584, *r* = 0.816, *p* = 0.092].

No significant changes in spasticity as assessed with the MAS were observed in the mid-PAS (*n* = 5, Md = 0.00 points), in the post-PAS (*n* = 5, Md = −2.00), or in the follow-up (*n* = 5, Md = 3,00) when compared with the corresponding values obtained in the pre-PAS [Supplementary Table 3, *n* = 5, χ^2^(3) = 3,065, *p* = 0.382; Friedman's test].

Differences between AIS sensory scores obtained in the pre-PAS and in the follow-up were non-significant (Supplementary Table 4, *n* = 5, Light touch Md = 1.00, *z* = −1.841, *p* = 0.066, Pin-prick Md = 0.00, *z* = −0.535, *p* = 0.593). SCIM scores collected in the pre-PAS and the follow-up did not differ (Supplementary Table 5, *n* = 5, Md = 0.00, *z* = −1.342, *p* = 0.180). Participants 4 and 5 improved their SCIM score by 15 and 9 points, respectively, mostly in Mobility subscales (items 10–17, Supplementary Table 6). The SCIM scores of the other participants remained the same. Participant 5 reported that pain disappeared in the right ankle after the PAS. Participants 1–4 did not have pain before or after treatment.

Participant 4 (with longest post-trauma time) reported slightly increased spasticity in his right hand and in the left leg as well as tiredness on week 2 of the intervention, which later disappeared. In week 5 he reported pain in his lower back while standing up or sitting down. The participant temporarily increased pain medication and received treatment from a physiatrist. The participant did not discontinue treatment. Other participants did not report any adverse effects.

## Discussion

The current study provides the first evidence that long-term PAS may increase leg muscle strength. We observed that long-term PAS applied for 2 months increased the total lower limb MMT score by ~1 point in each muscle. This effect was stable after a 1-month follow-up. The results of AIS motor score revealed a similar increase of muscle strength by ~1 point per muscle. Quantitatively similar findings were obtained in previous studies (16, 29), where long-term PAS was applied for 1 month and improved hand MMT scores by ~1 point in a group of individuals with chronic incomplete tetraplegia. Leg muscles are considerably larger than hand muscles and thus more profound changes than those in hand muscles are required to increase the MMT score (which evaluates the capacity, e.g., to lift the limb in different positions against gravity). Obtaining clinically meaningful results also requires greater changes in the lower than upper limbs. The similar increase of muscle strength observed in the current study can be explained by a longer intervention than that in our previous work that focused on upper limbs (16). The magnitude and stability of the overall PAS therapeutic effect can be positively related to the number of PAS sessions as observed in previous studies (14, 16, 17). However, age, severity of injury, and residual muscle strength can affect long-term PAS outcomes. Thus, it is important to consider these factors when planning individualized treatment to maximize the clinical efficacy of PAS.

In the present study, PAS was applied in a heterogenous group of participants. Initial lower limb muscle strength measured by MMT score and AIS motor score varied between participants. Stable improvements in lower limb muscle strength were observed in all participants. This increases the external validity of the present study. These results support efficacy of long-term PAS and also demonstrate an increase of walking speed post-PAS. Walking distance increased in 3 out of 4 ambulatory participants. The design of the study, which investigated both lower limb strength and locomotion, limited the possibility of stimulation of only one limb in all participants for controlling the effects of stimulation. However, in Participant 1, only the stimulated leg improved (Supplementary Table 1).

All participants had a standard rehabilitation schedule before the intervention and this schedule continued unchanged during the intervention and the follow-up period. The average changes of AIS motor scores during PAS ranged from 1.2 (in Mid-PAS) to 1.4 (in Post-PAS) points and were stable during follow-up. In SCI, motor improvements occur within the first 6–9 months and a plateau is usually reached by 12 months post-injury with minimal chance of further improvement (30). This excludes the possibility of spontaneous changes in muscle strength at the chronic stage. Improvements in muscle strength were also stable in follow-up as shown by MMT and AIS scores. The observed effects were not related to changes in spasticity or sensory function or caused by changes in training or medication; this is consistent with previous studies (14, 16, 29).

We selected the nerves innervating the weakest muscles for stimulation. This approach also led to improvements in the muscles that were not directly innervated by the stimulated nerves. Improvements in weak muscles plausibly affected the entire limb by making walking easier and thus promoting increased use of all muscles. Activation of the neighboring regions of the motor cortex and peripheral nerves is also plausible.

PAS was combined with a slight motor activation. Paired corticospinal-motoneuronal stimulation combined with a small level of isometric muscle contraction has been shown to significantly improve corticospinal transmission in people with SCI when compared with corresponding stimulation applied at rest (31). It is plausible that motor activation lowers motor thresholds centrally and peripherally; the number of orthodromic TMS-induced volleys and therefore the number of interactions between orthodromic and antidromic volleys might be increased. Movements might also activate secondary motor areas, rewiring them in synchrony with the stimulated corticospinal tract in a beneficial manner. The patients were instructed to activate the limbs during the stimulation only slightly; the amount of physical training added by this intervention when compared to their regular physiotherapy is small. Therefore, motor activation alone cannot explain the therapeutic effect.

A previous study revealed that high-frequency repetitive TMS applied immediately before robotic training sessions improved motor function in a chronic SCI patient (32). The authors hypothesized that rTMS increased M1 excitability and recruited stunned or dysfunctional connections. Our approach also incorporates both TMS and lower limb activation. However, PAS protocols, including ours, use low-frequency TMS, which are known to be inhibitory (33). This highlights the role of Hebbian plasticity as the most probable mechanism of the observed PAS effects (7, 8).

Correlation and significant linear relations between initial MMT scores collected in pre-PAS and changes of walking speed after PAS indicate that MMT scores have predictive value for changes in walking speed after long-term PAS. Key muscles can provide even better prediction. Changes in MMT scores in these muscles were shown to predict changes in walking speed after locomotor training (22). Regression analyses indicate that the same amount of stimulation will produce different effects on walking speed depending on the initial state of the leg muscles. Information on this relationship is particularly important for patient selection and estimation of treatment duration. Longer treatment plausibly produces larger improvements, according to the results of the previous PAS interventions (14, 16, 17). A previous study revealed the feasibility of achieving a maximal MMT score and independent use of upper extremities by means of long-term PAS (17). This opens new, attractive prospects in recovering locomotor function in people with chronic tetraplegia.

Age reduces neuroplasticity and the subsequent restoration of motor function after SCI (1), independent of rehabilitation strategy (22, 34). However, our results suggest that long-term PAS appears to be a promising therapeutic approach in 48–70-year-old subjects. Moreover, in this study we observed a stable increase of muscle strength in a highly heterogenous SCI group, including a non-ambulatory participant, individuals using a wheelchair, and independently walking subjects.

In our previous studies on PAS therapeutic effects in the upper limbs (14, 16, 17, 29), the ISI between TMS and PNS was calculated to coincide with stimulation-induced volleys in the cervical spinal cord, which is both the location of the stimulated lower motor neuron cell bodies and the site of the injury. Here, the stimuli were coinciding at the lumbar spinal cord, whereas the injury was in the cervical spinal cord. This suggests that PAS strengthens residual connectivity regardless of the location of the injury.

The 80–120-min duration of one PAS session depended on the number of stimulated nerves. The protocol was generally well-tolerated by the patients. Considering the variability of available resources in different countries, further research is needed to evaluate whether PAS protocols that employ higher TMS frequency with shorter duration (35, 36) could increase clinical feasibility.

A limitation of this study was the small sample size; the results of this case series should be considered exploratory. The observed motor changes and functional outcomes following long-term application of PAS in lower limbs enhance our understanding of the therapeutic mechanism of PAS and contribute to further development of long-term PAS therapy. The results of this proof-of-concept pilot study indicate a possible beneficial effect of long-term PAS in the rehabilitation of traumatic tetraplegia.

## Data Availability Statement

The raw data supporting the conclusions of this article will be made available by the authors, without undue reservation, to any qualified researcher.

## Ethics Statement

The studies involving human participants were reviewed and approved by The Ethics Committee of the Hospital District of Helsinki and Uusimaa. The participants provided their written informed consent to participate in this study.

## Author Contributions

AR, SS, EK, and AS data acquisition. AR, SS, EK, JM, and AS research design, analysis, and interpretation of data, drafting the work and revising it critically for important intellectual content, final approval of the version to be published, agreement to be accountable for all aspects of the work.

## Conflict of Interest

JM reports receiving travel expenses for lectures from Nexstim Inc outside the submitted work. The remaining authors declare that the research was conducted in the absence of any commercial or financial relationships that could be construed as a potential conflict of interest.
