# Supplementary material for: Effects of Long-Term Paired Associative Stimulation on Strength of Leg Muscles and Walking in Chronic Tetraplegia: A Proof-of-Concept Pilot Study
Source: Front Neurol. 2020 May 20;11:397. doi: 10.3389/fneur.2020.00397 (PMC7251052; doi:10.3389/fneur.2020.00397)
Supplement: Supplementary file 1 [file Table_1.pdf]

Supplementary table 1. Results of MMT of patient 1

| Nerve | Muscles | Left leg | | | | | | | Right leg | | | | | | |
|---|---|---|---|---|---|---|---|---|---|---|---|---|---|---|---|
| | | MMT score | | | | Difference | | | MMT score | | | | Difference | | |
| | | Pre-PAS | Mid-PAS | Post-PAS | F | Mid - Pre | Post - Pre | F - Pre | Pre-PAS | Mid-PAS | Post-PAS | F | Mid - Pre | Post - Pre | F - Pre |
| Gluteal | Gluteus maximus | 2 | 2 | 2 | 3 | 0 | 0 | 1 | 3 | 3 | 3 | 4 | 0 | 0 | 1 |
| Gluteal | Gluteus medius | 2 | 2 | 2 | 2 | 0 | 0 | 0 | 3 | 3 | 3 | 3 | 0 | 0 | 0 |
| Femoral | Iliopsoas | 4 | 5 | 5 | 4 | 1 | 1 | 0 | 5 | 5 | 5 | 5 | | | |
| Femoral | Quadriceps femoris | 5 | 5 | 5 | 5 | | | | 5 | 5 | 5 | 5 | | | |
| Peroneal | Tibialis anterior | 4 | 4 | 5 | 5 | 0 | 1 | 1 | 5 | 5 | 5 | 5 | | | |
| Peroneal | Long toe extensors | 5 | 5 | 5 | 5 | | | | 5 | 5 | 5 | 5 | | | |
| Tibial | Semitendinosus | 3 | 5 | 5 | 5 | 2 | 2 | 2 | 4 | 5 | 5 | 5 | 1 | 1 | 1 |
| Tibial | Gastrocnemius | 3 | 3 | 3 | 3 | 0 | 0 | 0 | 4 | 3 | 3 | 3 | -1 | -1 | -1 |
| Obturator | Adductors (hip) | 2 | 4 | 4 | 4 | 2 | 2 | 2 | 4 | 4 | 4 | 2 | 0 | 0 | -2 |

F – follow-up

Continuation of Supplementary table 1. Results of MMT of patient 2

| Nerve | Muscles | Left leg | | | | | | | Right leg | | | | | | |
|---|---|---|---|---|---|---|---|---|---|---|---|---|---|---|---|
| | | MMT score | | | | Difference | | | MMT score | | | | Difference | | |
| | | Pre-PAS | Mid-PAS | Post-PAS | F | Mid - Pre | Post - Pre | F - Pre | Pre-PAS | Mid-PAS | Post-PAS | F | Mid - Pre | Post - Pre | F - Pre |
| Gluteal | Gluteus maximus | 2 | 4 | 5 | 5 | 2 | 3 | 3 | 2 | 4 | 4 | 4 | 2 | 2 | 2 |
| Gluteal | Gluteus medius | 2 | 4 | 3 | 4 | 2 | 1 | 2 | 2 | 4 | 3 | 3 | 2 | 1 | 1 |
| Femoral | Iliopsoas | 3 | 5 | 5 | 5 | 2 | 2 | 2 | 2 | 5 | 4 | 4 | 3 | 2 | 2 |
| Femoral | Quadriceps femoris | 5 | 5 | 5 | 5 | | | | 5 | 5 | 5 | 5 | | | |
| Peroneal | Tibialis anterior | 5 | 5 | 5 | 5 | | | | 2 | 5 | 3 | 3 | 3 | 1 | 1 |
| Peroneal | Long toe extensors | 5 | 5 | 4 | 5 | | -1 | 0 | 4 | 4 | 4 | 3 | 0 | 0 | -1 |
| Tibial | Semitendinosus | 5 | 5 | 4 | 5 | | -1 | 0 | 3 | 4 | 5 | 5 | 1 | 2 | 2 |
| Tibial | Gastrocnemius | 3 | 4 | 3 | 4 | 1 | 0 | 1 | 2 | 3 | 3 | 3 | 1 | 1 | 1 |
| Other | Adductors (hip) | 5 | 5 | 5 | 5 | | | | 4 | 5 | 5 | 5 | 1 | 1 | 1 |

F – follow-up

Continuation of Supplementary table 1. Results of MMT of patient 3

| Nerve | Muscles | Left leg | | | | | | | Right leg | | | | | | |
|---|---|---|---|---|---|---|---|---|---|---|---|---|---|---|---|
| | | MMT score | | | | Difference | | | MMT score | | | | Difference | | |
| | | Pre-PAS | Mid-PAS | Post-PAS | F | Mid - Pre | Post - Pre | F - Pre | Pre-PAS | Mid-PAS | Post-PAS | F | Mid - Pre | Post - Pre | F - Pre |
| Gluteal | Gluteus maximus | 1 | 2 | 3 | 2 | 1 | 2 | 1 | 1 | 1 | 4 | 2 | 0 | 3 | 1 |
| Gluteal | Gluteus medius | 1 | 1 | 2 | 1 | 0 | 1 | 0 | 1 | 1 | 1 | 1 | 0 | 0 | 0 |
| Femoral | Iliopsoas | 1 | 2 | 3 | 2 | 1 | 2 | 1 | 1 | 2 | 1 | 2 | 1 | 0 | 1 |
| Femoral | Quadriceps femoris | 5 | 5 | 5 | 5 | | | | 5 | 5 | 5 | 5 | | | |
| Peroneal | Tibialis anterior | 5 | 5 | 5 | 3 | | 0 | -2 | 2 | 3 | 3 | 3 | 1 | 1 | 1 |
| Peroneal | Long toe extensors | n/a | n/a | n/a | n/a | | | | n/a | n/a | n/a | n/a | | | |
| Tibial | Semitendinosus | 1 | 2 | 2 | 2 | 1 | 1 | 1 | 1 | 2 | 2 | 2 | 1 | 1 | 1 |
| Tibial | Gastrocnemius | 3 | 3 | 3 | 5 | 0 | 0 | 2 | 2 | 3 | 3 | 5 | 1 | 1 | 3 |
| Other | Adductors (hip) | 1 | 2 | 3 | 3 | 1 | 2 | 2 | 1 | 2 | 3 | 2 | 1 | 2 | 1 |

F – follow-up, n/a – not available

Continuation of Supplementary table 1. Results of MMT of patient 4

| Nerve | Muscles | Left leg: MMT score | | | | Left leg: Difference | | | Right leg: MMT score | | | | Right leg: Difference | | |
|---|---|---|---|---|---|---|---|---|---|---|---|---|---|---|---|
| | | Pre-PAS | Mid-PAS | Post-PAS | F | Mid - Pre | Post - Pre | F - Pre | Pre-PAS | Mid-PAS | Post-PAS | F | Mid - Pre | Post - Pre | F - Pre |
| Gluteal | Gluteus maximus | 1 | 1 | 4 | 4 | 0 | 3 | 3 | 1 | 1 | 3 | 2 | 0 | 2 | 1 |
| Gluteal | Gluteus medius | 1 | 2 | 5 | 5 | 1 | 4 | 4 | 1 | 1 | 2 | 2 | 0 | 1 | 1 |
| Femoral | Iliopsoas | 1 | 1 | 4 | 5 | 0 | 3 | 4 | 1 | n/a | 1 | 2 | | 0 | 1 |
| Femoral | Quadriceps femoris | 5 | 5 | 5 | 5 | | | | 1 | 5 | 2 | 3 | 4 | 1 | 2 |
| Peroneal | Tibialis anterior | 5 | 5 | 5 | 5 | | | | 1 | 1 | 2 | 1 | 0 | 1 | 0 |
| Peroneal | Long toe extensors | 5 | 5 | 5 | 5 | | | | 1 | 1 | 3 | 3 | 0 | 2 | 2 |
| Tibial | Semitendinosus | 1 | 2 | 5 | 5 | 1 | 4 | 4 | 0 | 1 | 2 | 1 | 1 | 2 | 1 |
| Tibial | Gastrocnemius | 5 | 5 | 5 | 5 | | | | 1 | 1 | 3 | 1 | 0 | 2 | 0 |
| Other | Adductors (hip) | 2 | 2 | 2 | 4 | 0 | 0 | 2 | 1 | 2 | 2 | 2 | 1 | 1 | 1 |

F – follow-up, n/a – not available

Continuation of Supplementary table 1. Results of MMT of patient 5

| Nerve | Muscles | Left leg | | | | | | | Right leg | | | | | | |
|---|---|---|---|---|---|---|---|---|---|---|---|---|---|---|---|
| | | MMT score | | | | Difference | | | MMT score | | | | Difference | | |
| | | Pre-PAS | Mid-PAS | Post-PAS | F | Mid - Pre | Post - Pre | F - Pre | Pre-PAS | Mid-PAS | Post-PAS | F | Mid - Pre | Post - Pre | F - Pre |
| Gluteal | Gluteus maximus | 4 | 5 | 5 | 5 | 1 | 1 | 1 | 2 | 4 | 4 | 4 | 2 | 2 | 2 |
| Gluteal | Gluteus medius | 5 | 5 | 5 | 5 | | | | 3 | 4 | 4 | 4 | 1 | 1 | 1 |
| Femoral | Iliopsoas | 4 | 5 | 5 | 5 | 1 | 1 | 1 | 4 | 4 | 4 | 4 | 0 | 0 | 0 |
| Femoral | Quadriceps femoris | 5 | 5 | 5 | 5 | | | | 5 | 5 | 5 | 5 | | | |
| Peroneal | Tibialis anterior | 5 | 5 | 5 | 5 | | | | 4 | 5 | 5 | 5 | 1 | 1 | 1 |
| Peroneal | Long toe extensors | 5 | 5 | 5 | 5 | | | | 4 | 5 | 5 | 5 | 1 | 1 | 1 |
| Tibial | Semitendinosus | 5 | 5 | 5 | 5 | | | | 3 | 4 | 4 | 4 | 1 | 1 | 1 |
| Tibial | Gastrocnemius | 5 | 5 | 5 | 5 | | | | 4 | 4 | 5 | 5 | 0 | 1 | 1 |
| Other | Adductors (hip) | 5 | 5 | 5 | 5 | | | | 3 | 2 | 2 | 4 | -1 | -1 | 1 |

F – follow-up
