# Supplementary material for: Effects of Long-Term Paired Associative Stimulation on Strength of Leg Muscles and Walking in Chronic Tetraplegia: A Proof-of-Concept Pilot Study
Source: Front Neurol. 2020 May 20;11:397. doi: 10.3389/fneur.2020.00397 (PMC7251052; doi:10.3389/fneur.2020.00397)
Supplement: Supplementary file 2 [file Table_2.pdf]

Supplementary table 2. Results of ASIA motor test of patient 1

Patient 1	Motor score				Difference		
	Pre-PAS	Mid-PAS	Post-PAS	Follow-up	Mid-PAS - Pre-PAS	Post-PAS - Pre-PAS	Follow-up - Pre-PAS
Right leg							
Hip flexors	4	5	5	5	1	1	1
Knee extensors	5	5	5	5			
Ankle dorsiflexors	5	5	5	5			
Long toe extensors	5	5	5	5			
Ankle plantar flexors	5	5	5	5			
Left leg							
Hip flexors	3	4	4	4	1	1	1
Knee extensors	4	5	5	5	1	1	1
Ankle dorsiflexors	3	5	5	5	2	2	2
Long toe extensors	5	5	5	5			
Ankle plantar flexors	3	5	5	5	2	2	2

Continuation of Supplementary table 2. Results of ASIA motor of patient 2

Patient 2	Motor score				Difference		
	Pre-PAS	Mid-PAS	Post-PAS	Follow-up	Mid-PAS - Pre-PAS	Post-PAS - Pre-PAS	Follow-up - Pre-PAS
Right leg							
Hip flexors	4	5	5	5	1	1	1
Knee extensors	5	5	5	5			
Ankle dorsiflexors	1	4	3	4	3	2	3
Long toe extensors	4	5	5	4	1	1	0
Ankle plantar flexors	3	4	3	4	1	0	1
Left leg							
Hip flexors	4	5	5	5	1	1	1
Knee extensors	5	5	5	5			
Ankle dorsiflexors	5	5	5	5	0	0	0
Long toe extensors	5	5	4	5	0	-1	0
Ankle plantar flexors	4	4	3	4	0	-1	0

Continuation of Supplementary table 2. Results of ASIA motor of patient 3

Patient 3	Motor score				Difference		
	Pre-PAS	Mid-PAS	Post-PAS	Follow-up	Mid-PAS - Pre-PAS	Post-PAS - Pre-PAS	Follow-up - Pre-PAS
Right leg							
Hip flexors	1	1	1	2	0	0	1
Knee extensors	3	4	4	4	1	1	1
Ankle dorsiflexors	4	5	5	5	1	1	1
Long toe extensors	n/a	n/a	n/a	n/a	n/a	n/a	n/a
Ankle plantar flexors	2	5	5	5	3	3	3
Left leg							
Hip flexors	2	2	3	3	0	1	1
Knee extensors	5	4	5	5			
Ankle dorsiflexors	5	5	5	5			
Long toe extensors	n/a	n/a	n/a	n/a	n/a	n/a	n/a
Ankle plantar flexors	3	5	5	5	2	2	2

Continuation of Supplementary table 2. Results of ASIA motor of patient 4

Patient 4	Motor score				Difference		
	Pre-PAS	Mid-PAS	Post-PAS	Follow-up	Mid-PAS - Pre-PAS	Post-PAS - Pre-PAS	Follow-up - Pre-PAS
Right leg							
Hip flexors	2	2	3	2	0	1	0
Knee extensors	2	5	3	2	3	1	0
Ankle dorsiflexors	1	5	3	3	4	2	2
Long toe extensors	1	3	3	3	2	2	2
Ankle plantar flexors	2	3	5	3	1	3	1
Left leg							
Hip flexors	5	2	5	4			
Knee extensors	5	5	5	3			
Ankle dorsiflexors	5	5	5	5			
Long toe extensors	5	5	5	5			
Ankle plantar flexors	5	5	5	5			

Continuation of Supplementary table 2. Results of ASIA motor of patient 5

	Motor score				Difference		
Patient 5	Pre-PAS	Mid-PAS	Post-PAS	Follow-up	Mid-PAS - Pre-PAS	Post-PAS - Pre-PAS	Follow-up - Pre-PAS
Right leg							
Hip flexors	4	4	5	5	0	1	1
Knee extensors	4	4	5	4	0	1	0
Ankle dorsiflexors	1	1	5	4	0	4	3
Long toe extensors	3	3	5	5	0	2	2
Ankle plantar flexors	3	3	5	5	0	2	2
Left leg							
Hip flexors	5	5	5	5			
Knee extensors	5	5	5	5			
Ankle dorsiflexors	5	5	5	5			
Long toe extensors	5	5	5	5			
Ankle plantar flexors	5	5	5	5			
