# Supplementary material for: Effects of Long-Term Paired Associative Stimulation on Strength of Leg Muscles and Walking in Chronic Tetraplegia: A Proof-of-Concept Pilot Study
Source: Front Neurol. 2020 May 20;11:397. doi: 10.3389/fneur.2020.00397 (PMC7251052; doi:10.3389/fneur.2020.00397)
Supplement: Supplementary file 3 [file Table_3.pdf]

Supplementary table 3. Results of the Modified Asworth Scale

Patient	Asworth score (both legs)				Difference		
	Pre-PAS	Mid-PAS	Post-PAS	Follow-up	Mid-PAS - Pre-PAS	Post-PAS - Pre-PAS	Follow-up - Pre-PAS
1	15	16	18	18	1	3	3
2	6	6	3	10	0	-3	4
3	21	8	10	22	-13	-11	1
4	1	11	7	7	10	6	6
5	2	1	0	1	-1	-2	-1
Median	6.00	8.00	7.00	10.00	0.00	-2.00	3.00
Mean	9.00	8.40	7.60	11.60	-0.60	-1.40	2.60
SE	3.89	2.50	3.11	3.78	3.67	2.91	1.21
