# Supplementary material for: Effects of Long-Term Paired Associative Stimulation on Strength of Leg Muscles and Walking in Chronic Tetraplegia: A Proof-of-Concept Pilot Study
Source: Front Neurol. 2020 May 20;11:397. doi: 10.3389/fneur.2020.00397 (PMC7251052; doi:10.3389/fneur.2020.00397)
Supplement: Supplementary file 4 [file Table_4.pdf]

Supplementary table 4. ASIA sensory scores

Patient	ASIA sensory score (both legs)				Follow-up - Pre-PAS	
	Light touch		Pin-prick		Light touch	Pin-prick
	Pre-PAS	Follow-up	Pre-PAS	Follow-up		
1	18	32	16	32	14	16
2	14	16	11	11	2	0
3	16	17	1	2	1	1
4	12	12	9	9	0	0
5	27	28	24	18	1	-6
Median	16.00	17.00	11.00	11.00	1.00	0.00
Mean	17.40	21.00	12.20	14.40	3.60	2.20
SE	2.60	3.82	3.81	5.09	2.62	3.67
