# Supplementary material for: Effects of Long-Term Paired Associative Stimulation on Strength of Leg Muscles and Walking in Chronic Tetraplegia: A Proof-of-Concept Pilot Study
Source: Front Neurol. 2020 May 20;11:397. doi: 10.3389/fneur.2020.00397 (PMC7251052; doi:10.3389/fneur.2020.00397)
Supplement: Supplementary file 5 [file Table_5.pdf]

Supplementary table 5. Spinal Cord Independence Measure (SCIM)

Patient	SCIM score		Difference
	Pre-PAS	Follow-up	Follow-up - Pre-PAS
1	90	90	0
2	73	73	0
3	30	30	0
4	63	78	15
5	85	94	9
Median	73.00	78.00	0.00
Mean	68.20	73.00	4.80
SE	10.65	11.41	3.09
