# Supplementary material for: Effects of Long-Term Paired Associative Stimulation on Strength of Leg Muscles and Walking in Chronic Tetraplegia: A Proof-of-Concept Pilot Study
Source: Front Neurol. 2020 May 20;11:397. doi: 10.3389/fneur.2020.00397 (PMC7251052; doi:10.3389/fneur.2020.00397)
Supplement: Supplementary file 6 [file Table_6.pdf]

Supplementary table 6. Spinal Cord Independence Measure (SCIM) in participant 4 and 5

Item number	Item description	Participant 4		Participant 5	
		Pre-PAS	Follow-up	Pre-PAS	Follow-up
	Self-care				
1	Feeding	2	2	2	2
2a	Bathing (upper body)	2	2	1	1
2b	Bathing (lower body)	2	2	3	3
3a	Dressing (upper body)	2	3	4	4
3b	Dressing (lower body)	2	2	4	4
4	Grooming	2	2	3	3
	Respiration and Sphincter Management				
5	Respiration	10	10	10	10
6	Sphincter Management - Bladder	9	9	13	15
7	Sphincter Management - Bowel	5	8	10	8
8	Use of Toilet	4	4	5	5
	Mobility (room and toilet)				
9	Mobility in Bed and Action to Prevent Pressure Sores	6	6	6	6
10	Transfers: bed-wheelchair	1	2	2	2
11	Transfers: wheelchair-toilet-tub	1	2	2	2
	Mobility (indoors and outdoors, on even surface)				
12	Mobility Indoors	3	6	6	8
13	Mobility for Moderate Distances (10-100 meters)	6	6	5	8
14	Mobility Outdoors (more than 100 meters)	2	6	5	8
15	Stair Management	2	3	2	2
16	Transfers: wheelchair-car	1	2	2	2
17	Transfers: ground-wheelchair	1	1	0	1
	Total sum	63	78	85	94
